# Role of the Super-Enhancer Component Bromodomain Protein 4 in the Radiation Response of Human Head and Neck Squamous Cell Carcinoma Cells

**DOI:** 10.3390/cimb48010071

**Published:** 2026-01-10

**Authors:** Nanami Munakata, Hironori Yoshino, Masaharu Hazawa, Eichi Tsuruga

**Affiliations:** 1 Department of Radiation Science, Hirosaki University Graduate School of Health Sciences, Hirosaki 036-8564, Aomori, Japan; h24mh212@hirosaki-u.ac.jp (N.M.); tsuru@hirosaki-u.ac.jp (E.T.); 2 Institute for Frontier Science Initiative, Kanazawa University, Ishikawa 920-1192, Kanazawa, Japan; masaharu.akj@gmail.com

**Keywords:** radioresistance, bromodomain protein 4, human head and neck squamous cell carcinoma

## Abstract

Radiotherapy is an effective treatment for cancer; however, radioresistant cancer cells result in recurrence. Therefore, elucidating the mechanisms of radioresistance is urgently needed. Super-enhancers (SEs) are clusters of enhancers occupied by a high density of master transcription factors, mediators, and bromodomain protein BRD4. Recently, we reported that ΔNp63, an oncogenic transcription factor, promotes radioresistance in human head and neck squamous cell carcinoma (HNSCC) cells. As ΔNp63 establishes SEs in HNSCC cells, SEs may be involved in radioresistance. Here, we investigated the role of the SE component BRD4 in the radiation responses of HNSCC cells using a BRD4 degrader ARV-771 or BRD4 knockdown. First, Western blotting confirmed that ARV-771 decreased BRD4 protein expression. ARV-771 treatment resulted in reduced cell proliferation and enhanced apoptosis in irradiated HNSCC cells. Moreover, colony formation assays revealed that both ARV-771 and BRD4 knockdown enhanced the radiosensitivity of HNSCC cells, suggesting BRD4 contributes to the radioresistance of HNSCC cells. Furthermore, fluorescence immunostaining revealed distinct localization patterns of γH2AX, a marker of DNA double-strand breaks, compared with BRD4 and ΔNp63 in irradiated cells. Notably, ARV-771 and BRD4 knockdown decreased ΔNp63 and BRD4 protein expression, whereas ΔNp63 knockdown had minimal impact on BRD4 expression. Taken together, these findings suggest that BRD4-dependent maintenance of ΔNp63 expression may contribute, at least in part, to the regulation of radioresistance in HNSCC cells.

## 1. Introduction

Most head and neck cancers, collectively known as head and neck squamous cell carcinoma (HNSCC), arise from the mucosal epithelium in the oral cavity, pharynx, and larynx. HNSCC is highly aggressive and heterogeneous [1,2]. Curative treatment with surgery is frequently difficult because the head and neck region contains several vital organs responsible for essential physiological functions, as well as a dense concentration of bones, muscles, nerves, and blood vessels in a relatively small space.

Currently, various approaches are employed for managing HNSCC, including surgery, chemotherapy, radiation therapy, and photodynamic therapy [3]. Among these strategies, radiation therapy is highly effective for localized treatment and is one of the key therapeutic options for HNSCC. However, some cancer cells exhibit radioresistance, resulting in recurrence [4]. Therefore, elucidating the mechanisms of radioresistance and developing strategies to overcome it are crucial for improving prognosis.

We conducted a search for relevant molecular regulators to identify factors involved in radioresistance in HNSCC cells. Consequently, our laboratory recently reported that knockdown of ΔNp63 reduced clonogenic survival of irradiated cells and enhanced radiation-induced apoptosis, indicating a functional role of ΔNp63 in modulating radiation responses [5]. ΔNp63 is an isoform of p63, a member of the p53 tumor suppressor family, and the major isoform of p63 expressed in squamous cell carcinoma (SCC) is ΔNp63α [6]. ΔNp63α serves as an oncogene in SCC and promotes the early steps of SCC development [7]. Kudo et al. reported that ΔNp63α can transcriptionally repress p53 target genes, including BAX and p21, thereby attenuating p53-dependent DNA damage responses following ionizing radiation [8]. Notably, our findings suggest that ΔNp63 contributes to radioresistance even in HNSCC cells harboring mutant p53, suggesting the involvement of p53-independent survival mechanisms [5].

In addition, ΔNp63 is a master transcription factor and a super-enhancer (SE) component. SEs are clusters of enhancers that strongly induce gene expression [9]. They are characterized by histone H3K27 acetylation (H3K27ac), bromodomain protein 4 (BRD4), and the mediator complex [10]. Although typical enhancer regions range from 0.2 to 1 kb in length, SEs are formed when multiple transcription factors bind to the mediator complex at several adjacent enhancers, causing a large enhancer cluster [9]. Although the precise functions of SEs are not yet fully understood, they have gained attention as major regulatory elements impacting cell identity and disease pathogenesis, particularly in cancer [11,12,13]. For example, SE disruption in squamous cell carcinoma cells has been demonstrated to suppress tumorigenicity and metastasis in human HNSCC stem cells [14]. These findings suggest that SEs play a significant role in regulating cancer cell self-renewal and tumorigenesis and can function as potential therapeutic targets in cancer management. Furthermore, since ΔNp63 regulates HNSCC cell radioresistance, SEs may play a role in radioresistance. However, details remain unclear.

BRD4, a member of the bromodomain and extra-terminal domain (BET) protein family, is a key component of SEs. By interacting with acetylated lysine residues in histone proteins, it preferentially binds to active enhancers, contributing to chromatin remodeling and gene transcription [15]. BRD4 mainly promotes RNA polymerase II phosphorylation and mediates RNA polymerase II pausing and elongation [16]. Considering its role in oncogene regulation [17], BRD4 has become a crucial target for cancer therapy, resulting in the development of multiple BET inhibitors [18]. Recently, BET degradation inducers utilizing the ubiquitin–proteasome system, known as proteolysis-targeting chimeras (PROTAC), have been developed and are currently being investigated for clinical applications [19]. Here, we investigated the role of SEs in the radiation response of HNSCC cells using ARV-771 [20,21], a PROTAC BRD4 degrader.

## 2. Materials and Methods

### 2.1. Reagents

PBS(−) (Ca^2+^, Mg^2+^-free Dulbecco’s phosphate-buffered saline) and methanol were purchased from Wako Pure Chemical Industries (Osaka, Japan). Propidium iodide (PI), fetal bovine serum (FBS), and dimethyl sulfoxide (DMSO) were purchased from Sigma-Aldrich (St. Louis, MO, USA). ARV-771 was purchased from Med Chem Express (Monmouth Junction, NJ, USA). Anti-BRD4 rabbit antibody (#63759), anti-deltaN p63 (E6Q3O) rabbit antibody (##67825), anti-Glyceraldehyde–3–phosphate dehydrogenase (GAPDH) rabbit antibody (#5174), anti-p-histone H2A.X (γH2AX; #9718), horseradish peroxidase-conjugated anti-rabbit IgG antibody (#7074), horseradish peroxidase-conjugated anti-mouse IgG antibody (#7076), and AlexaFluor 488^®^-conjugated mouse IgG antibody (#4408) were purchased from Cell Signaling Technology Japan, K.K. (Tokyo, Japan). Anti-BRD4 rabbit antibody (Ab128874) was purchased from Abcam plc (Cambridge, UK), anti-γH2AX mouse antibody (JBW301) from Upstate Biotechnology, Inc. (Lake Placid, NY, USA), and CF™ 543-conjugated anti-mouse IgG (H + L) antibody (#20306) from Biotium (Fremont, CA, USA). Anti-human ΔNp63 rabbit antibody (#619001), FITC-Annexin V, and Annexin V binding buffer were purchased from BioLegend (San Diego, CA, USA). Lipofectamine^®^ RNAiMAX, Select Predesigned siRNA against the gene encoding ΔNp63 (#1: no. s16413, #2: no. s16411), BRD4 (no. s22120), and Silencer^®^ Select negative #1 Control siRNA (no. 4390843) were obtained from Thermo Fisher Scientific, Inc. (Waltham, MA, USA).

### 2.2. Cell Culture and Treatment

Human HNSCC cell lines SAS and Ca9-22 were purchased from RIKEN Bio-Resource Center (Tsukuba, Japan). Cells were cultured in Dulbecco’s Modified Eagle Medium high glucose (Wako) medium containing 10% FBS and 1% penicillin (100 units/mL)–streptomycin (100 μg/mL) (Wako) at 37 °C in a humidified atmosphere containing 5% CO_2_.

Cells were seeded in 35 mm culture dishes at a density of 5.0 × 10^4^ cells/dish and cultured for 2 days. Following incubation, the culture medium was removed, the medium containing DMSO or ARV-771 (final concentration, 1.0 μM) was added, and cells were further cultured for 24 h. The ARV-771–containing medium was removed, and cells were washed with PBS (−). ARV-771 was used at a final concentration of 1.0 μM, which was selected based on previous studies demonstrating efficient BRD4 degradation at this dose [20,21]. Following 1 h incubation, cells were irradiated with X-rays. At 24 h after irradiation, each cell was collected using 0.25% trypsin/Ethylenediaminetetraacetic acid (EDTA) (Wako), counted using the trypan blue dye exclusion method, and the cells were used for each analysis. For proliferative ability evaluation and apoptosis analysis, cells were collected 3 days after replacing cells, counted using the trypan blue dye exclusion method, reseeded onto 35 mm dishes at a density of 5.0 × 10^4^ cells/dish, and cultured for another 3 days.

### 2.3. SiRNA Transfection

Cells were seeded in 24-well plates at a density of 4.0 × 10^4^ cells and transfected with siRNA targeting ΔNp63 and BRD4 using Lipofectamine^®^ RNAiMAX Transfection Reagent (Invitrogen, Thermo Fisher Scientific, Waltham, MA, USA) according to the manufacturer’s protocol. Following 48 h transfection, cells were harvested for subsequent analysis. The final siRNA concentration was 10 nM.

### 2.4. In Vitro X-Ray Irradiation

Cells were irradiated using an X-ray generator (MBR-1520R-3; Hitachi, Ltd., Tokyo, Japan) at 450 mm from the focus and at a dose rate of 0.99–1.02 Gy/min (150 kVp; 20 mA; 0.5 mm Al filter and 0.3 mm Cu filter).

### 2.5. Colony Formation Assay

The irradiated cells were harvested after 24 h of culture and appropriate cell numbers were seeded onto 60 mm culture dishes (Sumitomo Bakelite Co., Ltd., Sumitomo Bakelite Co., Ltd., Tokyo, Japan) Colonies containing more than 50 cells were counted. The surviving fraction was calculated as previously described [22].

### 2.6. SDS-PAGE and Western Blotting

SDS-PAGE and Western blot analysis were performed as previously described [23]. Equal amounts of protein (about 2 μg per lane) were loaded onto SDS–polyacrylamide gels. The following primary antibodies were used: anti-ΔNp63 antibody (1:3000), anti-BRD4 antibody (1:3000), and anti-GAPDH antibody (1:4000). After overnight dilution at 4 °C, the membrane was reacted with a secondary antibody (1:10,000) for 1 h at 24 °C and detected using chemiluminescence with Clarity Western ECL Substrate (Bio-Rad Laboratories, Inc., Hercules, CA, USA). Images were captured using the iBright 1500 system (Thermo Fisher Scientific, Inc.). GAPDH was used as the loading control.

### 2.7. Analysis of Apoptosis

Apoptosis was analyzed using FITC-Annexin V and PI staining according to the manufacturer’s instructions. Briefly, cells were harvested, washed twice with PBS (−), centrifuged at 1200 rpm for 5 min at 24 °C, and suspended in 100 μL of Annexin V binding buffer. Five microliters of FITC-Annexin V (90 μg/mL) and PI (1 mg/mL) were added to the cell suspension, and cells were incubated for 15 min at 24 °C in the dark. After adding Annexin V binding buffer, samples were analyzed using a flow cytometer (CytoFLEX; Beckman-Coulter, Inc., Brea, CA, USA).

### 2.8. Fluorescence Immunostaining

SAS cells were seeded onto 35 mm dishes with submerged coverslips, irradiated with 2 Gy, and incubated for 30 min. Following incubation, cells were fixed in 4% paraformaldehyde (NACALAI TESQUE, INC., Kyoto, Japan) for 15 min at 24 °C. Following fixation, the coverslips were washed with PBS (−), and the samples were blocked with blocking buffer (5% bovine serum albumin/0.3% Triton X-100/PBS [−]) for 1 h. Subsequently, the samples were incubated overnight at 4 °C with the following primary antibody (1:200) in 1% bovine serum albumin/0.3% Triton X-100/PBS (−). Following incubation, the coverslips were washed with PBS (−), and a secondary antibody (1:200) in 1% bovine serum albumin/0.3% Triton X-100/PBS (−) was added and incubated for 1 h at 24 °C. Subsequently, the coverslips were washed with PBS (−), sealed, and mounted on glass slides using Prolong^®^ Gold Antifade Reagent with DAPI (#8961). The samples were analyzed using a confocal laser microscope (LSM 710; Carl Zeiss Co., Ltd., Tokyo, Japan).

### 2.9. Statistical Analysis

Data were presented as means ± standard error of three independent experiments. Comparison of the two groups was performed using Student’s t-test. A *p*-value of <0.05 was considered statistically significant. Statistical analysis was performed using Excel (Microsoft 365, Washington, DC, USA) with the add-in software Statcel4 (The Publishing OMS Ltd., Tokyo, Japan).

## 3. Results

### 3.1. Proliferation and Apoptosis in Non- or X-Ray–Irradiated HNSCC Cells Treated with ARV-771

**Figure 1:**
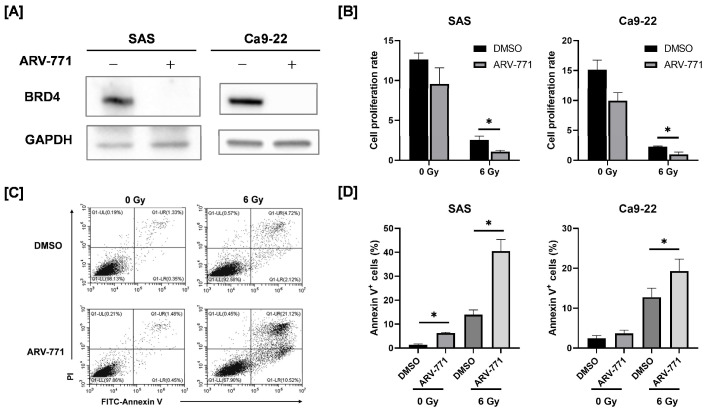
Effect of ionizing radiation on the proliferation and apoptosis of ARV-771–treated head and neck squamous cell carcinoma (HNSCC) cells. (**A**) ARV-771–treated SAS and Ca9-22 cells for 24 h were collected and analyzed for bromodomain protein 4 (BRD4) protein expression using Western blotting. (**B**–**D**) ARV-771–treated cells were irradiated with 6 Gy and cultured for 3 days. Cells were counted, reseeded, and cultured for another 3 days (total, 6 days). The cultured cells for 3 or 6 days were harvested for estimation of proliferation (**B**) and apoptosis analysis (**C**,**D**). (**B**) Results show the cell proliferation rate calculated from the ratio of the number of viable cells at 3 (for SAS) or 6 days (for Ca9-22) of culture to the number of seeded cells. * *p* < 0.05. (**C**) Representative cytogram of Annexin V/propidium iodide staining in SAS cells are shown. (**D**) Results are shown as percentage of Annexin V-positive cells. * *p* < 0.05.

We treated HNSCC cells with the BRD4 degrader ARV-771 and analyzed BRD4 protein expression to investigate the role of BRD4 in the cellular radiation response of HNSCC. ARV-771-treated cells exhibited lower BRD4 expression than DMSO-treated controls (Figure 1A).

Subsequently, we investigated cell proliferation and apoptosis in ARV-771–treated HNSCC cells under non- and X-ray–irradiated conditions. ARV-771 showed minimal impact on the proliferation rate of non-irradiated HNSCC cells (Figure 1B). However, 6 Gy–irradiated cells demonstrated significantly reduced proliferation at days 3 and 6 for SAS and Ca9-22 cells, respectively (Figure 1B). Moreover, apoptosis analysis revealed that ARV-771–treated cells had a higher percentage of apoptotic cells following 6 Gy irradiation than DMSO controls at the corresponding time points (Figure 1C,D).

### 3.2. Effects of ARV-771 or BRD4 Knockdown on Radiosensitivity of HNSCC Cells

**Figure 2:**
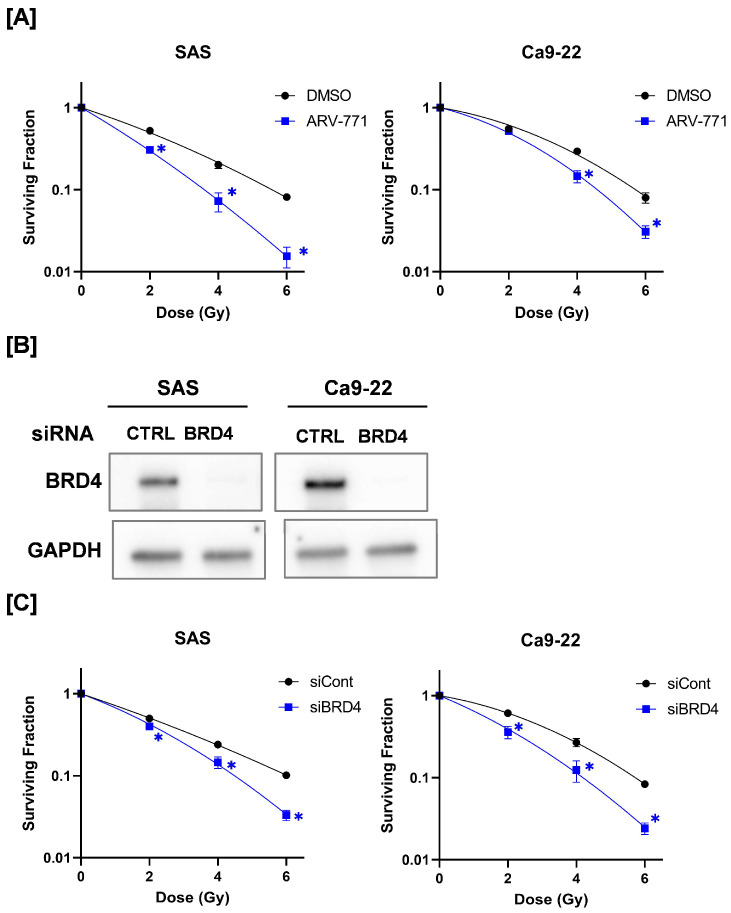
Effects of ARV-771 or BRD4 knockdown on radiosensitivity of HNSCC cells. (**A**) ARV-771–treated HNSCC cells were irradiated with X-rays, and cells were collected at 24 h following irradiation for colony assay. Results show survival rates at each dose with respect to non-irradiated cells. * *p* < 0.05. (**B**) SAS and Ca9-22 cells transfected with siRNA targeting BRD4 were harvested for BRD4 protein expression analysis by Western blotting. (**C**) BRD4-knockdown HNSCC cells were irradiated with X-rays, and cells were collected at 24 h following irradiation for colony assay. Results show survival rates at each dose with respect to non-irradiated cells. * *p* < 0.05.

We evaluated the radiosensitivity of ARV-771–treated HNSCC cells using a colony formation assay. As shown in Figure 2A, the surviving fractions of SAS and Ca9-22 cells were lower in the ARV-771-treated group than those in the DMSO-treated group, suggesting that ARV-771 enhances HNSCC cell radiosensitivity (Figure 2A). Similarly, BRD4 knockdown enhanced the radiosensitivity of HNSCC cells (Figure 2B,C). Taken together, these results indicate that BRD4 is involved in the radioresistance of HNSCC cells.

To evaluate whether ARV-771 or BRD4 knockdown affects radiosensitivity through alterations in cell cycle distribution, we performed cell cycle analysis following ARV-771 treatment and BRD4 knockdown. As shown in Supplementary Figure S1A, ARV-771 treatment increased the G2/M population in SAS cells, whereas it increased the G1 population in Ca9-22 cells. In contrast, BRD4 knockdown consistently increased the G1 population in both SAS and Ca9-22 cells (Supplementary Figure S1B). Importantly, an increase in the G1-phase population is generally associated with relative radioresistance compared with G2/M-phase cells [24]. Despite this, both ARV-771 treatment and BRD4 knockdown enhanced radiosensitivity in our experiments (Figure 2). These findings suggest that the observed changes in radiosensitivity cannot be sufficiently explained by alterations in cell cycle distribution alone.

### 3.3. Association of BRD4 with DNA Double-Strand Breaks (DSBs) in X-Ray–Irradiated SAS Cells

As DNA damage triggers radiation-induced cellular responses [22], we subsequently analyzed the association between DNA damage and BRD4. We investigated the localization of γH2AX and BRD4. γH2AX is widely used as a sensitive marker of DSBs; however, it also plays an essential role in the DNA damage response by serving as a platform for the recruitment and amplification of DNA repair and checkpoint signaling factors, including MDC1, 53BP1, and BRCA1 [25,26].

**Figure 3:**
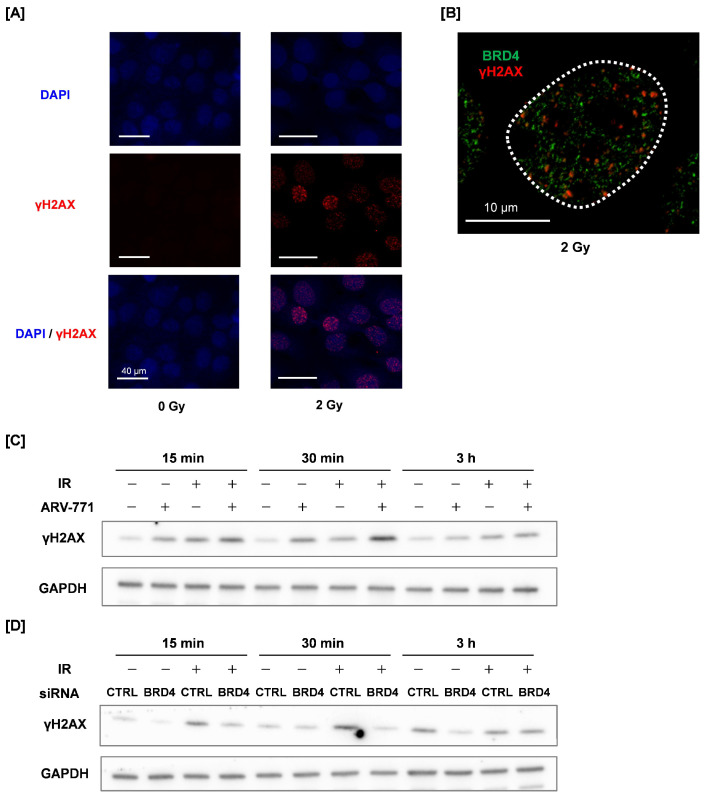
γH2AX and BRD4 localization and effects of ARV-771 or BRD4 knockdown γH2AX expression in irradiated SAS cells. (**A**,**B**) SAS cells were irradiated with 2 Gy and cultured for 30 min. Samples are analyzed using immunofluorescence staining. (**A**) DAPI (nuclei) and γH2AX in non-irradiated and 2 Gy–irradiated SAS cells. (**B**) Expression of BRD4 (green) and γH2AX (red) in 2 Gy–irradiated SAS cells. The white dotted line in the figure indicates the outline of the nucleus. (**C**,**D**) ARV-771–treated (**C**) or BRD4 knockdown (**D**) SAS cells were irradiated with X-rays, and cells were collected at 15 min, 30 min, and 3 h following irradiation for an analysis of γH2AX expression by Western blotting.

As shown in Figure 3A, γH2AX foci are observed in SAS cells at 30 min following 2 Gy irradiation, whereas they are rarely detected in non-irradiated cells. Notably, confocal laser microscopy analysis revealed that the signal distribution of γH2AX and BRD4 in 2 Gy–irradiated SAS cells was mutually exclusive (Figure 3B). This finding suggests that DSBs do not occur in the SE region. Therefore, it is suggested that the SE region is a DNA damage-resistant genomic region.

We evaluated the γH2AX levels by Western blotting when ARV-771 was combined with irradiation. ARV-771 treatment markedly enhanced radiation-induced γH2AX levels compared with irradiation alone (Figure 3C), indicating increased DNA damage accumulation when BRD4-mediated SE function was disrupted. These results suggest the notion that SE-associated chromatin states contribute to limiting DNA damage accumulation following irradiation. In addition, ARV-771 treatment alone increased basal γH2AX levels even in the absence of irradiation (Figure 3C), suggesting that BRD4 degradation induces endogenous DNA damage or enhances DNA damage signaling. Upon irradiation, ARV-771 further augmented γH2AX accumulation, consistent with an increased DNA damage burden and/or impaired resolution of DNA lesions. In contrast, BRD4 knockdown attenuated radiation-induced γH2AX induction (Figure 3D). Notably, the opposite effects of ARV-771 treatment and BRD4 knockdown on γH2AX accumulation suggest that pharmacological BRD4 degradation and genetic BRD4 depletion influence DNA damage responses through distinct mechanisms. These divergent effects indicate that γH2AX levels reflect not only DNA repair kinetics but also BRD4-dependent chromatin context and DNA damage signaling capacity.

### 3.4. Association Between BRD4 and ΔNp63 in Irradiated SAS Cells

**Figure 4:**
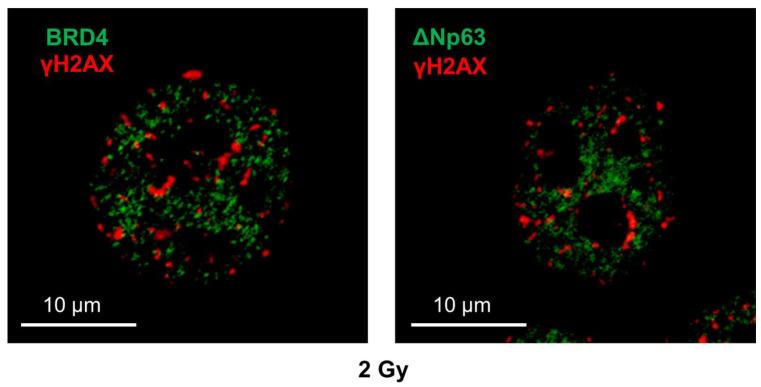
γH2AX and BRD4 or ΔNp63 localization in irradiated SAS cells. SAS cells were irradiated with 2 Gy and cultured for 30 min. Samples were analyzed using immunofluorescence staining. (**Left panel**) Expression of BRD4 (green) and γH2AX (red) in 2 Gy–irradiated cells. (**Right panel**) Expression of ΔNp63 (green) and γH2AX (red) in 2 Gy–irradiated cells.

We previously reported that the knockdown of ΔNp63, an SE component, improved HNSCC cell radiosensitivity [5]. Therefore, we analyzed the association between BRD4 and ΔNp63 in the irradiated SAS cells. Initially, we analyzed γH2AX and ΔNp63 localization. ΔNp63 and γH2AX, as well as BRD4 and γH2AX, exhibited different subnuclear localization in 2 Gy–irradiated SAS cells (Figure 4). This finding suggests that DSBs cannot occur in the regions where ΔNp63 or BRD4 is highly expressed in the irradiated cells.

**Figure 5:**
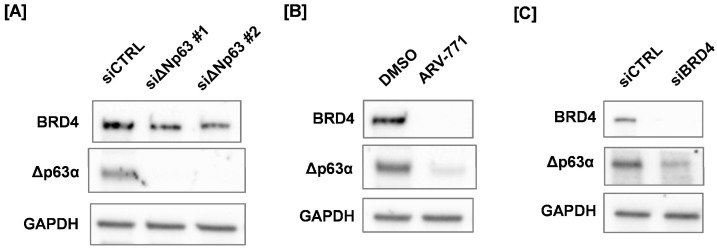
Protein expression of BRD4 and ΔNp63α treated with ΔNp63 knockdown, ARV-771, and BRD4 knockdown cells. (**A**) ΔNp63 knockdown, (**B**) ARV-771–treated, (**C**) BRD4 knockdown SAS cells were harvested for Western blotting to analyze BRD4 and ΔNp63α protein expression.

Next, we analyzed BRD4 and ΔNp63α protein expression in ΔNp63 knockdown or ARV-771-treated SAS cells. ΔNp63 knockdown effectively reduced ΔNp63α protein expression but had little effect on BRD4 protein levels (Figure 5A). In contrast, ARV-771 treatment reduced both BRD4 and ΔNp63α protein expression (Figure 5B). In addition, BRD4 knockdown resulted in efficient depletion of BRD4 and was accompanied by a reduction in ΔNp63α protein levels (Figure 5C). Taken together, these results suggest that BRD4 contributes to the maintenance of ΔNp63α expression.

## 4. Discussion

Elucidating the mechanisms of radioresistance and their regulation in cancer cells is essential for improving the efficacy of radiotherapy. In this study, we focused on BRD4, a key SE component, to elucidate the role of SEs in HNSCC cell radioresistance. Our findings showed that ARV-771–treated HNSCC cells with reduced BRD4 protein expression exhibited high radiosensitivity and low proliferation following irradiation. In addition, BRD4 knockdown enhanced radiosensitivity of HNSCC cells. These results suggest the role of BRD4 in HNSCC cell radioresistance. Furthermore, we noted that γH2AX foci were not observed at BRD4-enriched sites in irradiated cells, suggesting that radiation-induced DSBs do not occur in SE regions where BRD4 is highly expressed. Collectively, BRD4 may regulate HNSCC cell radioresistance by protecting the genomic regions involved in the survival from radiation.

Notably, γH2AX levels were differentially affected by pharmacological BRD4 degradation and BRD4 knockdown. ARV-771 increased basal γH2AX levels even in the absence of irradiation and further enhanced γH2AX accumulation after irradiation, whereas BRD4 knockdown attenuated radiation-induced γH2AX induction. These contrasting effects suggest that γH2AX reflects not only the extent of DNA double-strand breaks but also BRD4-dependent chromatin context and DNA damage signaling capacity. In this regard, BRD4 degradation by ARV-771 may induce endogenous DNA damage and/or potentiate DNA damage signaling by disrupting transcription-associated chromatin dynamics. Consistent with this interpretation, BRD4 has been shown to suppress R-loop accumulation and transcription–replication conflicts, and its loss results in increased DNA damage and γH2AX accumulation even in the absence of exogenous genotoxic stress [27]. In contrast, BRD4 knockdown may reduce γH2AX accumulation through distinct mechanisms, potentially involving transcriptional reprogramming of DNA damage response factors or adaptive changes in chromatin organization. Indeed, BRD4 regulates multiple aspects of genome maintenance beyond transcriptional control, including replication stress responses and chromatin-mediated DNA damage signaling pathways [17]. Thus, the divergent effects of BRD4 degradation and knockdown on γH2AX levels likely reflect fundamental differences in how BRD4 loss is achieved and how chromatin-associated DNA damage responses are engaged. At present, it remains unclear whether these effects represent direct modulation of DNA damage induction or indirect consequences of altered DNA damage response pathways, and additional analyses using complementary DNA damage indicators will be required to fully elucidate the mechanisms by which BRD4 regulates radiation responses.

Accumulating epigenomic evidence has revealed extensive reprogramming of SE landscapes in SCC, including HNSCC, leading to aberrant activation of oncogenic transcriptional programs that regulate tumor cell identity, stemness, and therapeutic resistance [28,29]. BRD4 has been shown to preferentially occupy SE regions in SCC models and to drive the expression of key oncogenes and lineage-defining transcription factors, such as TP63/ΔNp63 and MET [21,30]. Genome-wide enhancer profiling further demonstrates that p63 preferentially binds SEs and cooperates with chromatin regulators including BRD4 to sustain oncogenic transcriptional states in SCC cells [31,32]. SE-associated transcriptional programs have been implicated in aggressive tumor phenotypes, metastasis, and resistance to anticancer therapies, and BRD4 is frequently overexpressed in HNSCC, correlating with poor clinical outcomes [33]. Although direct clinical evidence linking SE enrichment or BRD4 genomic occupancy to radioresistance in HNSCC patients remains limited, these epigenomic observations collectively support a mechanistic framework in which BRD4-dependent SE function contributes to cellular responses to therapeutic stress, including ionizing radiation. Our findings extend this framework by providing functional evidence that disruption of BRD4-dependent SE activity enhances radiation-induced DNA damage responses in HNSCC cells.

Beyond chromatin-associated mechanisms, physical properties of SE-associated condensates may also influence DNA damage susceptibility. Recent evidence has indicated that liquid–liquid phase separation (LLPS) is involved in SE formation [34,35]. LLPS is a biological and physical phenomenon wherein a homogeneous liquid phase separates into two distinct liquid phases, similar to how oil and water separate. In biological systems, LLPS is involved in organizing cellular components without the necessity for membrane-bound organelles [36] and regulating cellular functions, including transcription [37]. We reported that the interior of LLPS condensates is a diluted aqueous environment [38]. Since low linear energy transfer (LET) radiation, including X-rays, induces DNA damage primarily through radicals generated by the radiolysis of water molecules, accounting for 60–70% of the damage [39], it is possible that X-rays cannot induce DSBs in SE regions formed by LLPS due to the lower water content. In contrast, high-LET radiation, including α-rays, can induce DNA damage independently of radical formation. Therefore, evaluating whether high-LET radiation can induce DNA damage in SE regions is necessary.

Similarly to BRD4, ΔNp63 localization was excluded from γH2AX-positive regions in irradiated cells, suggesting that genomic regions occupied by ΔNp63 may be relatively protected from radiation-induced DNA damage. We observed that ARV-771 treatment reduced ΔNp63α protein expression, whereas ΔNp63 knockdown had little effect on BRD4 protein levels. In addition, BRD4 knockdown was accompanied by a reduction in ΔNp63α expression. Consistent with these observations, we previously reported that ΔNp63 knockdown enhances radiation-induced apoptosis and radiosensitivity in HNSCC cells [5], which are also observed following ARV-771 treatment. Taken together, these findings suggest that BRD4-dependent maintenance of ΔNp63α expression may contribute, at least in part, to the regulation of radiation-induced apoptosis and radioresistance in HNSCC cells, although additional studies will be required to determine whether this relationship is direct or mediated through intermediate factors.

**Figure 6:**
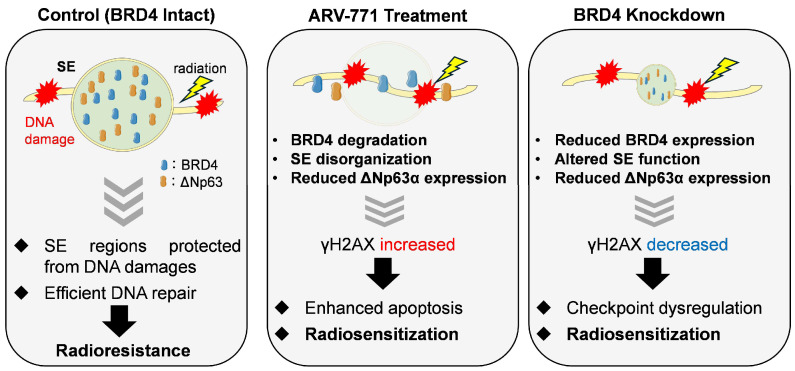
A proposed working model of BRD4-dependent regulation of DNA damage responses. Under a condition that BRD4 intact, BRD4-associated SEs support transcriptional programs involved in cell survival and DNA repair, contributing to efficient DNA damage repair and relative radioresistance. ARV-771 treatment induces pharmacological degradation of BRD4 and disorganization of SEs, leading to enhanced γH2AX signaling, transcriptional stress, and increased radiosensitization. In contrast, BRD4 knockdown alters SE function without inducing acute SE degradation, resulting in reduced γH2AX levels following irradiation and a distinct mode of radiosensitization. This model proposes that γH2AX reflects different biological processes depending on the mode of BRD4 suppression.

To integrate these observations, we propose a schematic model illustrating the differential effects of pharmacological BRD4 degradation and genetic BRD4 knockdown on SE integrity and DNA damage responses (Figure 6). Under basal conditions, BRD4 cooperates with ΔNp63 at SEs to sustain transcriptional programs involved in cell survival and DNA repair, thereby contributing to radioresistance. ARV-771–mediated BRD4 degradation induces rapid SE collapse, leading to transcriptional stress and aberrant DNA damage signaling, manifested as elevated γH2AX levels even in the absence of irradiation. In contrast, BRD4 knockdown partially preserves SE structures but disrupts the proper initiation and spatial organization of DNA damage response signaling, resulting in attenuated γH2AX induction following irradiation despite increased radiosensitivity. Thus, γH2AX reflects distinct underlying processes depending on the mode of BRD4 inhibition, highlighting the context-dependent role of BRD4 in coordinating SE architecture and DNA damage responses. This model is further supported by previous studies demonstrating a functional linkage between BRD4 and ΔNp63α in stratified squamous epithelial cells. Foffi et al. reported that BRD4 expression is positively correlated with p63 expression in human keratinocytes [40], supporting a role for BRD4 as an SE-associated co-activator of lineage-defining transcription factors. In this context, ARV-771–mediated BRD4 degradation may lead to reduced ΔNp63 expression by disrupting BRD4-dependent transcriptional programs.

The relevance of BRD4-dependent SE regulation may extend beyond HNSCC. Recently, we reported that ARV-771 enhances the radiosensitivity of human lung cancer cell line A549 [41]. Notably, A549 cells do not express ΔNp63 [42], suggesting that ARV-771 enhances A549 cell radiosensitivity independently of ΔNp63. In lung cancer cells, transcription factors, such as SMAD3, have been reported to constitute SEs [43,44]. Therefore, other SE-related transcription factors may be involved in radioresistance in lung cancer cells. As the transcription factors constituting SEs are different among cancer types, identifying those involved in radioresistance may result in reduced radioresistance. Notably, however, ARV-771 has been demonstrated to overcome radioresistance in some cancer types, thereby suggesting that targeting BRD4 can effectively regulate gene expression regardless of the specific transcription factor involved.

In conclusion, the results of this study suggest that the SE component BRD4 plays an important role in regulating radioresistance in HNSCC cells, potentially by protecting ge-nomic regions critical for cell survival from radiation-induced damage. Although the pre-cise molecular mechanisms linking BRD4-dependent SE function to DNA damage induction and repair remain to be fully clarified, our findings provide evidence that modulation of BRD4 alters cellular radiation responses. Further studies aimed at dissecting the mechanistic basis of SE-mediated radioresistance, incorporating additional DNA damage and repair assays as well as clinically relevant models, will be essential for establishing the therapeutic potential of targeting BRD4 to improve radiotherapy.

## Data Availability

The original contributions presented in this study are included in the article/Supplementary Material. Further inquiries can be directed to the corresponding author(s).

## Supplementary Materials

The following supporting information can be downloaded at: https://www.mdpi.com/article/10.3390/cimb48010071/s1. Reference [45] is cited in Supplementary Materials.

## Abbreviations

The following abbreviations are used in this manuscript:

SEs	Super-enhancers
BRD4	Bromodomain protein 4
HNSCC	Head and neck squamous cell carcinoma
BET	Bromodomain and extra-terminal domain
PI	Propidium iodide
FBS	Fetal bovine serum
DMSO	Dimethyl sulfoxide
DSBs	Double-Strand Breaks
LLPS	Liquid–liquid phase separation
LET	Linear energy transfer

## References

[1] Chen S.M.Y., Krinsky A.L., Woolaver R.A., Wang X., Chen Z., Wang J.H. (2020). Tumor immune microenvironment in head and neck cancers. Mol. Carcinog..

[2] Johnson D.E., Burtness B., Leemans C.R., Lui V.W.Y., Bauman J.E., Grandis J.R. (2020). Head and neck squamous cell carcinoma. Nat. Rev. Dis. Primers.

[3] Jiang Z., Yang X., Ainiwaer M., Chen F., Liu J. (2022). Recent clinical and preclinical advances in external stimuli-responsive therapies for head and neck squamous cell carcinoma. J. Clin. Med..

[4] Zhang H., Wang X., Ma Y., Zhang Q., Liu R., Luo H., Wang Z. (2023). Review of possible mechanisms of radiotherapy resistance in cervical cancer. Front. Oncol..

[5] Sato K., Yoshino H., Sato Y., Nakano M., Tsuruga E. (2023). ΔNp63 regulates radioresistance in human head and neck squamous carcinoma cells. Curr. Issues Mol. Biol..

[6] Cancer Genome Atlas Network (2015). Comprehensive genomic characterization of head and neck squamous cell carcinomas. Nature.

[7] Devos M., Gilbert B., Denecker G., Leurs K., Mc Guire C., Lemeire K., Hochepied T., Vuylsteke M., Lambert J., Van Den Broecke C. (2017). Elevated ΔNp63α levels facilitate epidermal and biliary oncogenic transformation. J. Investig. Dermatol..

[8] Kudo K.I., Tsuyama N., Nagata K., Imaoka T., Iizuka D., Sugai-Takahashi M., Muramatsu M., Sakai A. (2022). ΔNp63α transcriptionally represses p53 target genes involved in the radiation-induced DNA damage response: ΔNp63α may cause genomic instability in epithelial stem cells. Radiat. Oncol..

[9] Whyte W.A., Orlando D.A., Hnisz D., Abraham B.J., Lin C.Y., Kagey M.H., Rahl P.B., Lee T.I., Young R.A. (2013). Master transcription factors and mediator establish super-enhancers at key cell identity genes. Cell.

[10] Bacabac M., Xu W. (2023). Oncogenic super-enhancers in cancer: Mechanisms and therapeutic targets. Cancer Metastasis Rev..

[11] Chu L.Y., Wu F.C., Fang W.K., Hong C.Q., Huang L.S., Zou H.Y., Peng Y.H., Chen H., Xie J.J., Xu Y.W. (2023). Secreted proteins encoded by super enhancer-driven genes could be promising biomarkers for early detection of esophageal squamous cell carcinoma. Biomed. J..

[12] Jia Q., Deng H., Wu Y., He Y., Tang F. (2023). Carcinogen-induced super-enhancer RNA promotes nasopharyngeal carcinoma metastasis through NPM1/c-Myc/NDRG1 axis. Am. J. Cancer Res..

[13] Teng S., Li Y.E., Yang M., Qi R., Huang Y., Wang Q., Zhang Y., Chen S., Li S., Lin K. (2020). Tissue-specific transcription reprogramming promotes liver metastasis of colorectal cancer. Cell Res..

[14] Dong J., Li J., Li Y., Ma Z., Yu Y., Wang C.Y. (2021). Transcriptional super-enhancers control cancer stemness and metastasis genes in squamous cell carcinoma. Nat. Commun..

[15] Cheung K.L., Kim C., Zhou M.M. (2021). The functions of BET proteins in gene transcription of biology and diseases. Front. Mol. Biosci..

[16] Yang Z., Yik J.H.N., Chen R., He N., Jang M.K., Ozato K., Zhou Q. (2005). Recruitment of P-TEFb for stimulation of transcriptional elongation by the bromodomain protein Brd4. Mol. Cell.

[17] Donati B., Lorenzini E., Ciarrocchi A. (2018). BRD4 and cancer: Going beyond transcriptional regulation. Mol. Cancer.

[18] Filippakopoulos P., Qi J., Picaud S., Shen Y., Smith W.B., Fedorov O., Morse E.M., Keates T., Hickman T.T., Felletar I. (2010). Selective inhibition of BET bromodomains. Nature.

[19] To K.K.W., Xing E., Larue R.C., Li P.-K. (2023). BET Bromodomain inhibitors: Novel design strategies and therapeutic applications. Molecules.

[20] Hu J., Yang J., Zhou R., Chen K., Zhao H., Zhou Y. (2025). Discovery of a potent BRD4 PROTAC and evaluation of its bioactivity in breast cancer cell lines. Biochem. Pharmacol..

[21] Raina K., Lu J., Qian Y., Altieri M., Gordon D., Rossi A.M., Wang J., Chen X., Dong H., Siu K. (2016). PROTAC-induced BET protein degradation as a therapy for castration-resistant prostate cancer. Proc. Natl. Acad. Sci. USA.

[22] Yoshino H., Iwabuchi M., Kazama Y., Furukawa M., Kashiwakura I. (2018). Effects of retinoic acid-inducible gene-I-like receptors activations and ionizing radiation cotreatment on cytotoxicity against human non-small cell lung cancer in vitro. Oncol. Lett..

[23] Yoshino H., Kumai Y., Kashiwakura I. (2017). Effects of endoplasmic reticulum stress on apoptosis induction in radioresistant macrophages. Mol. Med. Rep..

[24] Pawlik T.M., Keyomarsi K. (2004). Role of cell cycle in mediating sensitiveity to radiotherapy. Int. J. Radiat. Oncol. Biol. Phys..

[25] Stucki M., Jackson S.P. (2006). γH2AX and MDC1: Anchoring the DNA-damage-response machinery to broken chromosomes. DNA Repair.

[26] Bonner W.M., Redon C.E., Dickey J.S., Nakamura A.J., Sedelnikova O.A., Solier S., Pommier Y. (2008). γH2AX and cancer. Nat. Rev. Cancer.

[27] Lam F.C., Kong Y.W., Huang Q., Vu Han T.L., Maffa A.D., Kasper E.M., Yaffe M.B. (2020). BRD4 prevents the accumulation of R-loops and protects against transcription-replication collision events and DNA damage. Nat. Commun..

[28] Yi M., Tan Y., Wang L., Cai J., Li X., Zeng Z., Xiong W., Li G., Li X., Tan P. (2020). TP63 links chromatin remodeling and enhancer reprogramming to epidermal differentiation and squamous cell carcinoma development. Cell Mol. Life Sci..

[29] Zhou R.W., Parsons R.E. (2023). Etiology of super-enhancer reprogramming and activation in cancer. Epigenetics Chromatin.

[30] Fisher M.L., Balinth S., Hwangbo Y., Wu C., Ballon C., Wilkinson J.E., Goldberg G.L., Mills A.A. (2021). BRD4 Regulates Transcription Factor ΔNp63α to Drive a Cancer Stem Cell Phenotype in Squamous Cell Carcinomas. Cancer Res..

[31] Riege K., Kretzmer H., Sahm A., McDade S.S., Hoffmann S., Fischer M. (2020). Dissecting the DNA binding landscape and gene regulatory network of p63 and p53. eLife.

[32] Zamuner F.T., Chan S.S., Kessler M.D., Vorontsov I.E., Loginov A., Erbe R., Imada E., Xie D.X., Guo T., Fertig E.J. (2025). Epigenetic profiling reveals key super-enhancer networks driving oncogenesis in HPV-positive HNSCC. iScience.

[33] Wu Y., Wang Y., Diao P., Zhang W., Li J., Ge H., Song Y., Li Z., Wang D., Liu L. (2019). Therapeutic Targeting of BRD4 in Head Neck Squamous Cell Carcinoma. Theranostics.

[34] Sabari B.R., Dall’Agnese A., Boija A., Klein I.A., Coffey E.L., Shrinivas K., Abraham B.J., Hannett N.M., Zamudio A.V., Manteiga J.C. (2018). Coactivator condensation at super-enhancers links phase separation and gene control. Science.

[35] Hazawa M., Ikliptikawati D.K., Iwashima Y., Lin D.-C., Jiang Y., Qiu Y., Makiyama K., Matsumoto K., Kobayashi A., Nishide G. (2023). Super-enhancer trapping by the nuclear pore via intrinsically disordered regions of proteins in squamous cell carcinoma cells. Cell Chem. Biol..

[36] Peng P.H., Hsu K.W., Wu K.J. (2021). Liquid–liquid phase separation (LLPS) in cellular physiology and tumor biology. Am. J. Cancer Res..

[37] Nozawa R.S., Yamamoto T., Takahashi M., Tachiwana H., Maruyama R., Hirota T., Saitoh N. (2020). Nuclear microenvironment in cancer: Control through liquid-liquid phase separation. Cancer Sci..

[38] Hazawa M., Amemori S., Nishiyama Y., Iga Y., Iwashima Y., Kobayashi A., Nagatani H., Mizuno M., Takahashi K., Wong R.W. (2021). A light-switching pyrene probe to detect phase-separated biomolecules. iScience.

[39] Zhang Y., Martin S.G. (2014). Redox proteins and radiotherapy. Clin. Oncol. J..

[40] Foffi E., Violante A., Pecorari R., Lena A.M., Rugolo F., Melino G., Candi E. (2024). BRD4 sustains p63 transcriptional program in keratinocytes. Biol. Direct.

[41] Matsumoto K., Ikliptikawati D.K., Makiyama K., Mochizuki K., Tobita M., Kobayashi I., Voon D.C.-C., Lim K., Ogawa K., Kashiwakura I. (2024). Phase-separated super-enhancers confer an innate radioresistance on genomic DNA. J. Radiat. Res..

[42] Hazawa M., Yoshino H., Nakagawa Y., Shimizume R., Nitta K., Sato Y., Sato M., Wong R.W., Kashiwakura I. (2020). Karyopherin-β1 regulates radioresistance and radiation-increased programmed death-ligand 1 expression in human head and neck squamous cell carcinoma cell lines. Cancers.

[43] Zhang T., Xia W., Song X., Mao Q., Huang X., Chen B., Liang Y., Wang H., Chen Y., Yu X. (2022). Super-enhancer hijacking LINC01977 promotes malignancy of early-stage lung adenocarcinoma addicted to the canonical TGF-β/SMAD3 pathway. J. Hematol. Oncol..

[44] Jie X., Fong W.P., Zhou R., Zhao Y., Zhao Y., Meng R., Zhang S., Dong X., Zhang T., Yang K. (2021). USP9X-mediated KDM4C deubiquitination promotes lung cancer radioresistance by epigenetically inducing TGF-β2 transcription. Cell Death Differ..

[45] Sato K., Yoshino H., Sato Y., Sasaki F., Munakata N., Tsuruga E. (2025). Impact of the ATM/Chk2 pathway and cell cycle phase on radiation-induced senescence in A549 human lung cancer cells. Biomed Rep..

