# Supplementary material for: Role of the Super-Enhancer Component Bromodomain Protein 4 in the Radiation Response of Human Head and Neck Squamous Cell Carcinoma Cells
Source: Curr Issues Mol Biol. 2026 Jan 10;48(1):71. doi: 10.3390/cimb48010071 (PMC12840072; doi:10.3390/cimb48010071)
Supplement: Supplementary file 1 [file cimb-48-00071-s001.zip › cimb-3882425-supplementary.pdf]

Supplementary Figure S1

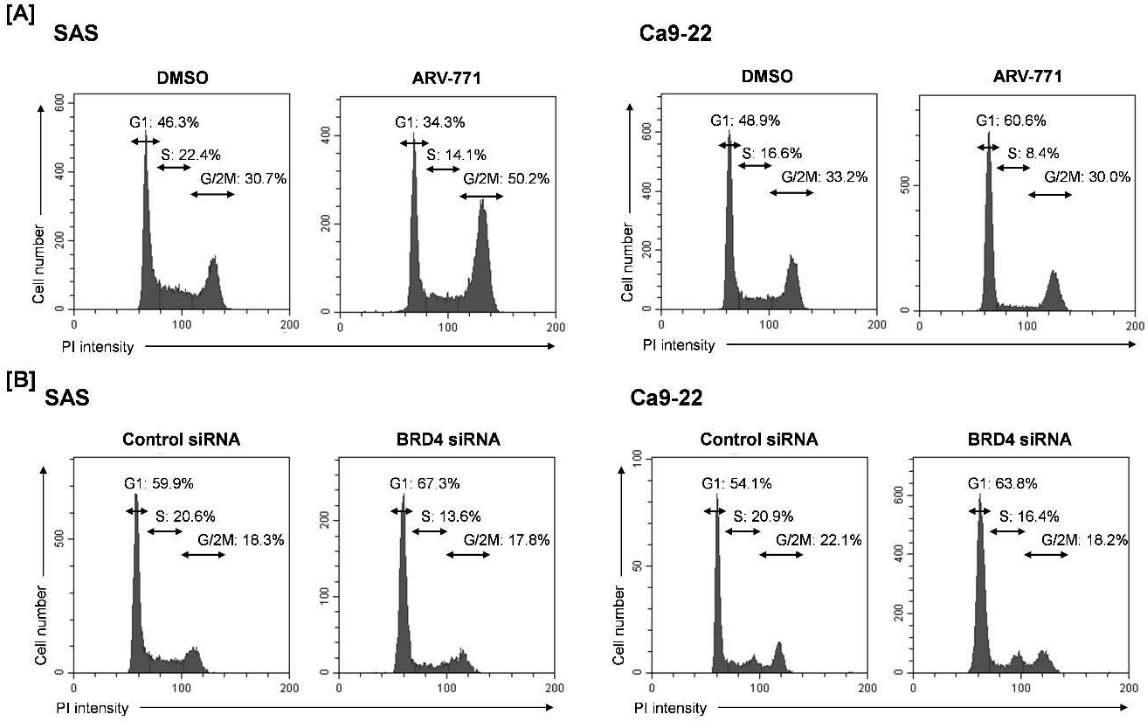

Supplementary Figure S1. Effects of ARV-771 or BRD4 knockdown on the cell-cycle distribution of HNSCC cells. (A, B) The cell-cycle distribution of ARV-771–treated (A) or BRD4 knockdown (B) HNSCC cells was analyzed by propidium iodide (PI) staining using a flow cytometer (CytoFLEX; Beckman Coulter), as previously reported [1]. Representative cell-cycle histograms are shown. The inset numbers indicate the proportion of cells in each cell-cycle phase.

Reference

1. Sato K, Yoshino H, Sato Y, Sasaki F, Munakata N, Tsuruga E. Impact of the ATM/Chk2 pathway and cell cycle phase on radiation-induced senescence in A549 human lung cancer cells. Biomed Rep. 2025;23(5):169.
